# La mucormycose rhino-orbito-cérébrale : ne pas méconnaître l’examen direct!

**DOI:** 10.48327/mtsi.v6i1.2026.702

**Published:** 2026-01-20

**Authors:** Imane ZOUAOUI, BAY Yassine BAY, Houda DOSS-BENNANI, Sarra AOUFI

**Affiliations:** 1 Laboratoire central de parasitologie-mycologie du Centre hospitalier universitaire Ibn Sina Rabat, Maroc; 2 Service d’ophtalmologie, hôpital des spécialités de Rabat, Maroc; 3 Faculté de médecine et de pharmacie de Rabat, Maroc; 4 Université Mohammed V de Rabat, Maroc

**Keywords:** Mucorales, Diabète, Infection fongique invasive, Infection opportuniste, Examen mycologique, Amphotéricine B, Maroc, Afrique du Nord, Mucorales, Diabetes, Invasive fungal infection, Opportunistic infection, Mycological examination, Amphotericin B, Morocco, North Africa

## Abstract

**Introduction:**

La mucormycose rhino-orbito-cérébrale est la forme clinique la plus fréquente des mucormycoses. Le diabète représente la pathologie sous-jacente la plus souvent en cause dans cette forme, surtout en cas de déséquilibre glycémique.

**Observation:**

Le cas rapporté concerne une adolescente de 13 ans, diabétique de type 1, admise pour une décompensation acidocétosique. Les examens cliniques et radiologiques ont révélé une cellulite orbitaire évolutive (stade II puis V de Chandler). Un traitement antibiotique et antifongique a été instauré mais sans amélioration. Une intervention chirurgicale a permis de retirer un tissu nécrosé dont les examens mycologique et anatomopathologique ont confirmé le diagnostic de mucormycose. La patiente a été traitée avec succès par amphotéricine B liposomale pendant deux mois.

**Discussion/Conclusion:**

Bien que peu fréquente, cette infection doit être systématiquement suspectée en cas de cellulite faciale, essentiellement chez un patient diabétique mal équilibré, afin de garantir une prise en charge rapide et efficace.

## Abstract (en)

**Introduction:**

Rhino-orbito-cerebral mucormycosis is the most common clinical form of mucormycosis. The most common underlying condition associated with this form is diabetes, especially in cases of glycemic imbalance.

**Observation:**

This report concerns a 13-year-old girl with type 1 diabetes who was admitted for ketoacidosis. Clinical and radiological examinations revealed progressive orbital cellulitis (Chandler stage II, then V). Despite initiating antibiotic and antifungal treatment, there was no improvement. Surgery was performed to remove necrotic tissue, and mycological and anatomopathological examinations confirmed the diagnosis of mucormycosis. The patient was successfully treated with liposomal amphotericin B for two months.

**Discussion/Conclusion:**

Although this infection is rare, it should always be suspected in cases of facial cellulitis, especially in patients with poorly controlled diabetes, to ensure rapid and effective treatment.

## Introduction

La mucormycose rhino-orbito-cérébrale (MROC) est une infection fongique invasive causée par des champignons appartenant à l’ordre des Mucorales [4]. Il s’agit d’une infection opportuniste grave, potentiellement mortelle, survenant principalement chez les patients immunodéprimés ou présentant des troubles métaboliques, notamment un diabète sucré mal équilibré [1,4]. Environ 70 % des patients atteints de MROC sont diabétiques, faisant du diabète le principal facteur de risque identifié pour cette affection [10].

Bien que l’incidence de cette maladie ait augmenté ces dernières années, notamment durant la pandémie de Covid-19 [23], elle reste encore mal connue et peu décrite dans notre pays.

Ce travail rapporte un cas de MROC illustrant les défis diagnostiques et thérapeutiques rencontrés et le rôle central du laboratoire dans la prise en charge.

## Observation

Nous rapportons le cas d’une jeune fille de 13 ans, connue diabétique de type 1 depuis l’âge de 6 ans, sous insulinothérapie avec un mauvais équilibre glycémique et des antécédents d’hospitalisations multiples pour décompensation acido-cétosique (DAC). Elle a été admise pour la prise en charge d’un nouvel épisode de DAC.

Trois mois avant cette admission, la patiente a présenté des épistaxis unilatérales droites récidivantes, survenant une à deux fois par semaine, sans autre symptôme ORL associé.

**Figure 1:**
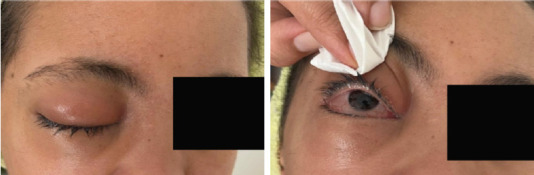
Tuméfaction périorbitaire droite + signes inflammatoires locaux

**Figure 2:**
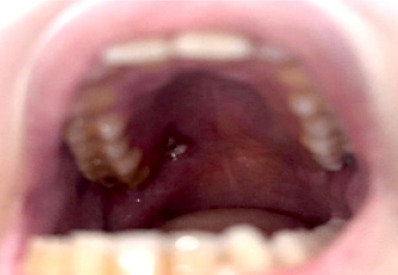
Nécrose + enduit blanchâtre au niveau du palais droit

L’examen clinique à son admission avait retrouvé une patiente fébrile, polypnéique à 42 cycles/ minute, avec des signes de déshydratation, nécessitant la mise en route urgente d’une réhydratation avec surveillance des paramètres vitaux. Par ailleurs, l’examen a révélé la présence d’une tuméfaction périorbitaire droite avec des signes inflammatoires locaux en regard (Fig. 1) et un aspect d’enduit blanchâtre recouvrant la partie droite du palais (Fig. 2).

Un bilan biologique a été fait objectivant:

- hyperleucocytose à 19 500 éléments/mm^3^ (N : 4 000-10 000), à prédominance neutrophile 15 300 éléments/mm3 (N : 1 500-7 000);
- thrombocytose à 419 000 éléments/mm^3^ (N : 150 000-400 000);
- hémoglobine à 8,6 g/dl (N : 13-16,5 g/dl);
- protéine C réactive à 151 mg/l (N < 5 mg/l);
- sérologies VIH, VHB, VHC et syphilis revenues négatives.

Sur le plan radiologique, le scanner orbitaire avait objectivé une cellulite préseptale droite stade I de Chandler complétée par une IRM orbito-cérébrale requalifiant la cellulite orbitaire en stade Il de Chandler, révélant une névrite optique et une encéphalite associée à une rhino-sinusite.

Devant ce tableau clinico-biologique et radiologique, le diagnostic de cellulite orbitaire d’origine rhino-sinusienne a été retenu et la lésion du palais a été attribuée à une candidose palatine.

La patiente a été mise sous traitement associant trois antibiotiques et un antifongique (céphalosporine de 3^e^ génération, métronidazole, amikacine et fluconazole) après une gestion de la décompensation cétoacidosique par une réhydratation massive et une insulinothérapie horaire.

Un écouvillonnage des lésions orbitaire et palatine a été réalisé en vue d’une analyse microbiologique. L’examen cytobactériologique du prélèvement avait isolé *Klebsiella pneumoniae* et l’examen mycologique du même échantillon était négatif. L’évolution après huit jours a été marquée par l’aggravation de sa lésion palatine avec perforation et issue de liquide par la narine droite.

Un 2^e^ scanner orbitaire et des sinus a été réalisé. Il a montré une cellulite orbitaire stade II de Chandler responsable d’une exophtalmie grade I + sinusite maxilo-ethmoido-sphénoidale.

Un avis ophtalmologique et neurologique a recommandé la poursuite du traitement par antibiothérapie et la surveillance de la patiente.

**Figure 3:**
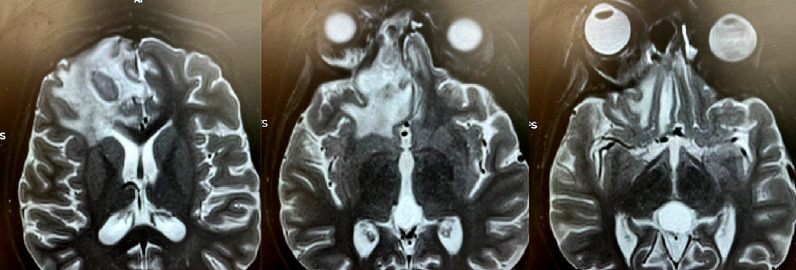
Scanner cérébral. Abcès frontal droit, IRM orbito-cérébrale, cellulite orbitaire droite compliquée d’une thrombose veineuse du sinus caverneux homolatéral et d’abcès cérébraux basifrontaux droits (stade V de Chandler)

Vingt jours plus tard, la patiente a présenté des céphalées intenses avec des crises convulsives ayant nécessité la réalisation d’un scanner cérébral montrant un abcès frontal droit. En complément, une IRM orbito-cérébrale a été réalisée objectivant une cellulite orbitaire droite compliquée d’une thrombose veineuse du sinus caverneux homolatéral et d’abcès cérébraux basifrontaux droits type V de Chandler (Fig. 3).

Une nécrosectomie étendue, accompagnée d’une méatotomie et d’une éthmoïdectomie totale, a été réalisée au bloc opératoire. Un tissu nécrosé, composé d’éléments graisseux et cartilagineux, a été réséqué et adressé au laboratoire de parasitologie-mycologie.

**Figure 4:**
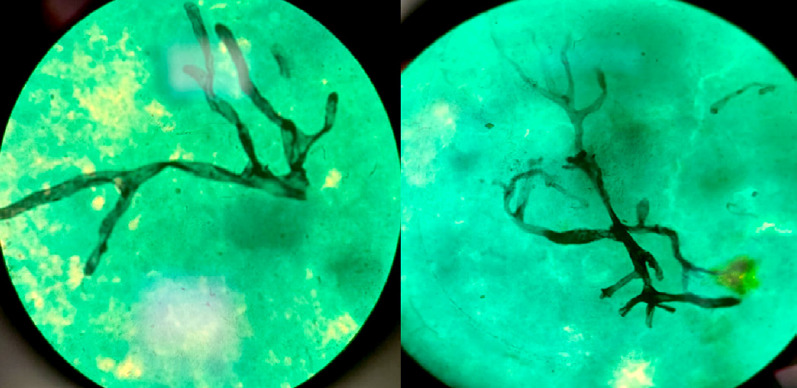
Mucorale : Aspect microscopique (observé au microscope optique après coloration au Gomori-Grocott au grossissement 1000x à l’immersion)

L’examen direct à l’état frais et après coloration au Gomori-Grocott a mis en évidence de nombreux filaments mycéliens, rubanés, non septés, ayant souvent des ramifications à angles droits, en faveur des Mucorales (Fig. 4). La culture sur les différents milieux de Sabouraud pendant un mois est revenue négative.

L’examen anatomopathologique a permis de confirmer le diagnostic de mucormycose surin-fectée. Il a révélé une muqueuse nasale largement ulcérée en surface, recouverte d’un tissu de granulation polymorphe et d’un enduit fibrino-leucocytaire riche en polynucléaires altérés, avec par endroits la présence de filaments mycéliens épais, ramifiés et présentant des bifurcations centrales caractéristiques. Des foyers de nécrose suppurée ainsi que de nombreux amas de germes banals ont également été observés.

La patiente a été mise sous amphotéricine B liposomale à raison de 3 mg/kg en intraveineux après un délai de 14 jours entre l’établissement du diagnostic et le début du traitement, en raison de l’indisponibilité du médicament et de la nécessité d’une demande et d’une procédure administrative spécifique.

L’évolution a été globalement favorable sur l’ensemble des plans clinique, biologique et radiologique. Toutefois, la patiente a gardé une cécité séquellaire de l’œil droit.

La durée du traitement par amphotéricine B a été de 60 jours, les antifongiques oraux de relais, tels que le posaconazole et l’isavuconazole, n’étant pas disponibles au Maroc. La patiente bénéficie actuellement d’une surveillance multidisciplinaire rapprochée en ORL, en ophtalmologie, en neurochirurgie et en endocrinologie. Aucune rechute n’a été rapportée à ce jour (huit mois de suivi).

## Discussion

Les Mucorales sont considérées comme la troisième cause la plus fréquente d’infections fongiques invasives chez les patients immunodéprimés, après les espèces de *Candida* et d’*Aspergillus*, et sont associées à une morbidité et mortalité élevées [6].

Autrefois considérée comme une infection rare, la mucormycose a connu une augmentation significative de son incidence à l’échelle mondiale au cours des deux dernières décennies [10,15]. Cette recrudescence a été particulièrement marquée durant la pandémie de Covid-19, au point d’être qualifiée d’ « épidémie dans la pandémie », en raison de l’augmentation sans précédent des cas signalés dans de nombreux pays [9].

Bien que son incidence reste variable selon les régions, elle est estimée entre 0,005 et 1,7 cas par million d’habitants dans le monde [25]. Toutefois, certains pays affichent des taux nettement plus élevés. C’est notamment le cas de l’Inde, où la prévalence de la mucormycose atteint environ 140 cas par million d’habitants, soit près de 80 fois la moyenne mondiale [10,15,23]. Cette incidence est principalement liée à la prévalence élevée du diabète mal contrôlé et à l’impact de la pandémie de Covid-19. D’autres facteurs ont été cités comme la tuberculose, les traitements immunosuppresseurs ou la corticothérapie [27]. Sur le continent africain, la mucormycose demeure rarement rapportée [10]. Une revue couvrant la période de 1960 à 2022 n’a identifié que 408 cas dans 12 des 54 pays africains, révélant ainsi une importante pénurie de données. Les cas signalés provenaient majoritairement d’Afrique du Nord, suivie de l’Afrique australe, tandis que les autres sous-régions restaient très peu documentées. L’Égypte représentait à elle seule plus des deux tiers des cas rapportés [26].

Dans la région Moyen-Orient et Afrique du Nord (MENA), une analyse combinée des données issues du registre FungiScope^®^ et de la littérature scientifique (période 1990-2019) a permis d’identifier 310 cas de mucormycose, avec une augmentation progressive au fil des décennies. L’Iran est le pays ayant rapporté le plus grand nombre de cas (74), suivi de la Tunisie (49), du Liban et de l’Arabie Saoudite (28 chacun), de l’Égypte (20), de l’Irak (11) et du Qatar (10). D’autres pays, dont le Maroc, ont rapporté moins de 10 cas et aucun cas n’a été signalé en Algérie, en Libye, en Syrie ou au Yémen [32].

Au Maroc, une revue de la littérature fondée sur les bases de données PubMed et le registre national des thèses Toubkal, a permis d’identifier 25 cas de mucormycose publiés entre 2000 et 2024 [2,3,5,7,8,11-13,16-19,21,22,24,29-31,33]. En y incluant le cas rapporté dans la présente étude, le total s’élève à 26 cas. La faible incidence apparente pourrait s’expliquer par une sous-notification, des retards diagnostiques ou un accès limité aux outils diagnostiques modernes.

Parallèlement aux évolutions médicales, le profil des populations à risque de mucormycose s’est progressivement élargi. Si le diabète sucré représentait historiquement le principal facteur de risque, l’usage croissant de traitements immunosuppresseurs (chimiothérapie, immunothérapie), ainsi que la multiplication des transplantations d’organes solides et de greffes de cellules souches hématopoïétiques, ont entraîné l’émergence de nouveaux contextes de vulnérabilité [1,28]. Plus récemment, l’infection à SARS-CoV-2, en particulier dans les formes sévères nécessitant une corticothérapie prolongée, a été identifiée comme un facteur de risque supplémentaire de mucormycose [10,23]. Néanmoins, dans les régions où l’accès aux soins reste limité, le diabète demeure le facteur de risque prédominant, notamment dans les formes rhinoorbito-cérébrales [1,4,23].

Les manifestations cliniques des mucormycoses sont variées mais résultent dans tous les cas de la capacité des Mucorales à induire des nécroses tissulaires *via* leur pouvoir angio-invasif. Cinq formes cliniques majeures sont décrites : rhino-orbito-cérébrale, pulmonaire, cutanée, gastro-intestinale et disséminée. La forme rhino-orbito-cérébrale constitue la forme la plus fréquente représentant 30 à 50 % des cas de mucormycoses [2,4,6,28]. Au niveau national, cette forme représente également la présentation clinique la plus fréquente, avec 19 cas recensés sur un total de 26 cas de mucormycose signalés [2,3,5,7,8,11-13,16-19,21,22,24,29-31,33].

Elle évolue rapidement en trois phases successives en cas de retard diagnostique, d’abord la phase naso-sinusienne, suivie de la phase orbitaire et en fin la phase cérébrale.

Les principaux signes cliniques au début sont naso-sinusiens. La brièveté et la banalité de ces signes risquent de faire méconnaître le diagnostic à un stade précoce [1,9]. Ce fut le cas de notre patiente où l’épistaxis est passée inaperçue, ce qui a entraîné son admission à un stade tardif. De plus, la symptomatologie à l’admission (tuméfaction périorbitaire) prêtait à confusion : le diagnostic initial évoquait une cellulite orbitaire d’origine rhino-sinusienne, tandis que la lésion palatine avait été attribuée à une candidose.

L’association d’une atteinte orbitaire et sinusienne et la présence de lésions nécrotiques au niveau des muqueuses est en faveur de mucormycose [4]. Il est donc judicieux d’évoquer cette pathologie devant toute tuméfaction périorbitaire inexpliquée, précédée de signes naso-sinusiens, en particulier chez les patients diabétiques, notamment ceux en DAC.

Sur le plan radiologique, La réalisation d’un scanner permet d’affirmer à la fois la sinusite par épaississement de la muqueuse sinusienne et l’atteinte osseuse. L’IRM, complémentaire du scanner, explore les atteintes oculaires, cérébrales, les extensions vers le sinus caverneux et les thromboses ou anévrismes intracrâniens [28]. La confirmation diagnostique repose sur l’examen mycologique et/ou anatomopathologique montrant des filaments mycéliens irréguliers, non septés [1,4,14].

Divers prélèvements peuvent être envisagés (biopsies, écouvillonnage ou aspiration de pus) [6], mais les biopsies tissulaires sont considérées comme les échantillons de choix [14,15,28].

L’examen mycologique (examen direct et culture) constitue la méthode diagnostique de référence bien que la culture soit fréquemment négative [6,14]. À l’examen direct, les filaments des Mucorales présentent classiquement un aspect large, mesurant entre 6 et 15 µm de diamètre. Ils apparaissent hyalins, irréguliers, non cloisonnés et parfois déformés [20]. La seule observation microscopique de ces filaments caractéristiques ne reste que présomptive d’infection à Mucorales [20]. Aucune identification d’espèce n’est bien sûr possible en l’absence de culture. Rappelons que les Mucorales ne poussent généralement pas dans les milieux de Sabouraud additionnés d’actidione. L’examen anatomopathologique (ou histopatho-logique) vient en complément pour conforter le diagnostic et rapporter la preuve du caractère invasif de la mycose [6]. Cependant, il n’est pas considéré comme adapté à un diagnostic précoce en raison de sa faible sensibilité [14].

Récemment, des outils moléculaires comme la PCR ou la spectrométrie de masse MALDI-TOF se sont révélés très prometteurs pour l’identification des Mucorales en permettant un diagnostic beaucoup plus précoce et rapide [6,28]. Cependant, leur disponibilité est encore limitée.

Le traitement des MROC reste difficile, et la rapidité de sa mise en place conditionne le pronostic. Une approche multidisciplinaire constitue la base du traitement, associant le contrôle de la pathologie sous-jacente, notamment l’équilibre d’un diabète décompensé, suivi d’un débridement chirurgical des tissus infectés et d’un traitement antifongique adapté [6]. L’amphotéricine B est la molécule de choix [1,4,10,15,23,28].

## Conclusion

Cette observation illustre les conséquences d’un retard dans le diagnostic et la prise en charge thérapeutique de la mucormycose. Malgré la rareté de cette infection, elle doit être suspectée devant toute cellulite faciale en particulier chez le patient diabétique mal contrôlé pour assurer une prise en charge clinique et biologique rapide et rigoureuse.

## Consentement de la patiente

Le consentement de la patiente a été obtenu pour la publication de cet article.

## Financement

Cette étude n’a reçu aucun financement.

## Contributions des auteurs et autrices

Imane ZOUAOUI : Conception de l’étude, prospection bibliographique, collecte, analyse et interprétation des données, rédaction du manuscrit Yassine BAY BAY : Réalisation d’une partie de l’étude (exécution des tests de laboratoire, recueil des données)

Houda DOSS-BENNANI : Réalisation d’une partie de l’étude (recueil des données, suivi clinique de la patiente, surveillance thérapeutique)

Sarra AOUFI : Approbation de la version finale

## Déclaration de liens d’intérêts

Aucun lien d’intérêt n’a été déclaré.
