# Supplementary material for: Rhino-orbito-cerebral mucormycosis: don’t overlook direct examination!
Source: Med Trop Sante Int. 2026 Jan 20;6(1):mtsi.v6i1.2026.702. [Article in French] doi: 10.48327/mtsi.v6i1.2026.702 (PMC13216903; doi:10.48327/mtsi.v6i1.2026.702)
Supplement: Related texts

# Research article: Rhino-orbito-cerebral mucormycosis: don’t overlook direct examination!

## Introduction

Rhino-orbito-cerebral mucormycosis (ROCM) is an invasive fungal infection caused by fungi in the order Mucorales [4]. This serious, potentially fatal opportunistic infection mainly occurs in immunocompromised patients or those with metabolic disorders, particularly poorly controlled diabetes mellitus [1,4]. Approximately 70% of ROCM patients are diabetic, making diabetes the main identified risk factor for this condition [10]. Although the incidence of this disease has increased in recent years, particularly during the SARS-CoV-2 pandemic [23], it remains poorly understood and rarely reported in our country. This paper reports a case of ROCM, illustrating the diagnostic and therapeutic challenges encountered, as well as the central role of the laboratory in its management.

## Case report

We present the case of a 13-year-old girl with a history of type 1 diabetes since age six. She was on insulin therapy with poor glycemic control and had been hospitalized multiple times for diabetic ketoacidosis (DKA). She was admitted for management of a new episode of DKA.

Three months prior to this admission, the patient experienced recurrent unilateral right epistaxis, occurring once or twice a week, with no other associated ear, nose, and throat symptoms.

Upon admission, the patient was found to be febrile, polypneic at 42 cycles per minute, and dehydrated, requiring urgent rehydration and monitoring of vital signs. The examination also revealed right periorbital swelling with local inflammatory signs (Fig. 1) and a whitish coating on the right side of the palate (Fig. 2).

Laboratory tests were performed and revealed the following:

**Figure 1:** Right periorbital swelling + local inflammatory signs

**Figure 2:** Necrosis + whitish coating on the right palate

- Hyperleukocytosis at 19,500 cells/mm³ (normal range: 4,000–10,000), with 15,300 neutrophils (normal range: 1,500–7,000);
- Thrombocytosis at 419,000 cells/mm³ (normal range: 150,000–400,000);
- Hemoglobin at 8.6 g/dL (normal range: 13–16.5 g/dL).
- C-reactive protein was 151 mg/L (normal range <5 mg/L).
- HIV, HBV, HCV, and syphilis serology tests were negative.

The orbital CT scan revealed stage I Chandler’s right preseptal cellulitis. An orbital-cerebral MRI reclassified the cellulitis as stage II Chandler’s and revealed optic neuritis and encephalitis associated with rhinosinusitis.

Based on these clinical, biological, and radiological findings, the diagnosis was orbital cellulitis of rhinosinusogenic origin, and the palatal lesion was attributed to candidiasis.

After managing ketoacidotic decompensation through massive rehydration and hourly insulin therapy, the patient was placed on a combination of three antibiotics and one antifungal agent (third-generation cephalosporin, metronidazole, amikacin, and fluconazole).

Swabs of the orbital and palatal lesions were taken for microbiological analysis.

Cytological and bacteriological examinations of the sample isolated *Klebsiella pneumoniae*; however, the mycological examination of the same sample was negative.

Eight days later, her condition worsened, resulting in perforation of the palatal lesion and fluid discharge from the right nostril.

A second orbital and sinus CT scan was performed. It showed stage II Chandler orbital cellulitis, causing grade I exophthalmos and maxillary, ethmoid, and sphenoid sinusitis.

Following an ophthalmological and neurological consultation, it was recommended that the patient continue antibiotic treatment and be monitored. Twenty days later, the patient presented with severe headaches and seizures, prompting a brain CT scan. The scan revealed a right frontal abscess. An orbital-cerebral MRI was also performed and revealed right orbital cellulitis complicated by venous thrombosis of the ipsilateral cavernous sinus and Chandler type V right basifrontal cerebral abscesses (Fig. 3).

**Figure 3:** Brain CT scan. Right frontal abscess, orbito-cerebral MRI, right orbital cellulitis complicated by venous thrombosis of the ipsilateral cavernous sinus and right basifrontal cerebral abscesses (Chandler stage V)

Extensive necrosectomy, accompanied by a meatotomy and total ethmoidectomy, was performed in the operating room. The necrotic tissue, which contained fatty and cartilaginous elements, was removed and sent to the parasitology-mycology laboratory.

Direct examination of the fresh tissue, as well as examination after Gomori-Grocott staining, revealed numerous mycelial filaments that were ribbon-like, non-septate, and often had right-angled branches. These characteristics are consistent with Mucorales (Fig. 4). Culture on various Sabouraud media for one month was negative.

**Figure 4:** Mucorale: Microscopic appearance (observed under an optical microscope after Gomori-Grocott staining at 1000x magnification with immersion)

Pathological examination confirmed the diagnosis of superinfected mucormycosis. The examination revealed extensive ulceration of the nasal mucosa, covered with polymorphic granulation tissue and a fibrin-leukocyte coating rich in altered polymorphonuclear cells. Thick, branched mycelial filaments with characteristic central bifurcations were also observed. Foci of suppurative necrosis and numerous clusters of common bacteria were also observed.

The patient was placed on liposomal amphotericin B at a dose of 3 mg/kg intravenously, with a 14-day delay between diagnosis and treatment initiation due to drug unavailability and the necessity of a specific request and administrative procedure. The patient had a favorable overall clinical, biological, and radiological outcome. However, the patient remained blind in her right eye.

Treatment with amphotericin B lasted 60 days because oral antifungal drugs, such as posaconazole and isavuconazole, are unavailable in Morocco. She is currently undergoing close monitoring by multidisciplinary teams from the departments of otolaryngology, ophthalmology, neurosurgery, and endocrinology. No relapse has been reported to date (after eight months of follow-up).

## Discussion

Mucorales are the third most common cause of invasive fungal infections in immunocompromised patients after *Candida* and *Aspergillus* species. They are associated with high morbidity and mortality [6].

Once considered rare, mucormycosis has seen a significant increase in incidence worldwide over the last two decades [10,15]. This resurgence has been particularly pronounced during the ongoing SARS-CoV-2 pandemic, with some describing it as an “epidemic within the pandemic” due to the unprecedented increase in reported cases in many countries [9].

Its incidence varies by region, but is estimated to be between 0.005 and 1.7 cases per million people worldwide [25]. However, some countries have significantly higher rates. India has one of the highest rates, with around 140 cases per million inhabitants, nearly 80 times the global average [10,15,23]. This high incidence is mainly due to the prevalence of poorly controlled diabetes and the impact of the pandemic. Other cited factors include tuberculosis, immunosuppressive treatments, and corticosteroid therapy [27].

Mucormycosis remains rarely reported on the African continent [10]. A review of cases from 1960 to 2022 identified only 408 instances across 12 of the 54 African countries, highlighting a substantial lack of data. Most of the reported cases came from North Africa, followed by Southern Africa; the other subregions were poorly documented. Egypt alone accounted for over two-thirds of the reported cases [26].

A combined analysis of data from the FungiScope^®^ registry and scientific literature from the Middle East and North Africa (MENA) region (1990-2019) identified 310 cases of mucormycosis, showing a gradual increase over the decades. Iran reported the most cases (74), followed by Tunisia (49), Lebanon and Saudi Arabia (28 each), Egypt (20), Iraq (11), and Qatar (10). Other countries, including Morocco, reported fewer than ten cases. No cases were reported in Algeria, Libya, Syria, or Yemen [32].

A review of PubMed databases and the Toubkal national thesis registry in Morocco identified 25 cases of mucormycosis published between 2000 and 2024 [2,3,5,7,8,11-13,16-19,21,22,24,29-31,33]. Including the case reported in this study, the total rises to 26 cases. The apparent low incidence could be explained by under-reporting, delays in diagnosis, or limited access to modern diagnostic tools.

Alongside medical advances, the population at risk for mucormycosis has gradually broadened. While diabetes mellitus has historically been the main risk factor, the increasing use of immunosuppressive treatments (e.g., chemotherapy and immunotherapy) and the growing number of solid organ and hematopoietic stem cell transplants have created new contexts of vulnerability [1,28]. Recently, SARS-CoV-2 infection, especially severe cases requiring prolonged corticosteroid therapy, has been identified as an additional risk factor for mucormycosis [10,23].

Nevertheless, in regions where access to care is limited, diabetes is the primary risk factor, especially for rhino-orbito-cerebral forms [1,4,23]. The clinical manifestations of mucormycosis vary, but all result from Mucorales’ ability to induce tissue necrosis via angioinvasion. There are five major clinical forms: rhino-orbito-cerebral, pulmonary, cutaneous, gastrointestinal, and disseminated. Rhino-orbito-cerebral mucormycosis is the most prevalent form, accounting for 30 to 50% of cases [2,4,6,28]. At the national level, it is also the most common clinical presentation, accounting for 19 out of 26 reported cases of mucormycosis [2,3,5,7,8,11-13,16-19,21,22,24,29-31,33].

In cases of delayed diagnosis, it progresses rapidly in three successive phases: first, the naso-sinus phase; then, the orbital phase; and finally, the cerebral phase.

The main clinical signs at onset are naso-sinus. The brevity and banality of these signs may lead to misdiagnosis in the early stages [1,9]. This occurred with our patient, whose epistaxis went unnoticed, resulting in late-stage admission. Additionally, the symptoms upon admission (periorbital swelling) were confusing; the initial diagnosis was orbital cellulitis of rhinosinusitis origin, and the palatal lesion was attributed to candidiasis.

The combination of orbital and sinus involvement, as well as the presence of necrotic lesions in the mucous membranes, is consistent with mucormycosis [4]. Therefore, it is advisable to consider mucormycosis in cases of unexplained periorbital swelling preceded by naso-sinus signs, particularly in diabetic patients with DKA.

A CT scan can radiologically confirm sinusitis due to thickening of the sinus mucosa and bone involvement. MRI, which complements CT scans, can be used to investigate ocular and cerebral involvement, as well as extension to the cavernous sinus. MRI can also detect intracranial thrombosis or aneurysms [28].

Diagnostic confirmation is based on a mycological and/or anatomopathological examination that shows irregular, non-septate mycelial filaments [1,4,14].

Various samples may be considered, such as biopsies, swabs, or pus aspiration [6], but tissue biopsies are considered the sample of choice [14,15,28].

The gold standard diagnostic method is mycological examination (direct examination and culture), although cultures are often negative [6,14]. Direct examination reveals that Mucorales filaments are typically broad, measuring between 6 and 15 µm in diameter. They appear hyaline, irregular, non-septate, and sometimes deformed [20]. However, microscopic observation of these characteristic filaments alone provides only presumptive evidence of Mucorales infection [20]. Of course, species identification is not possible without culturing. Note that Mucorales generally do not grow in Sabouraud media supplemented with actidione.

An anatomopathological (or histopathological) examination is used to confirm the diagnosis and demonstrate the invasive nature of the mycosis [6]. However, it is not suitable for early diagnosis due to its low sensitivity [14].

Recently, molecular tools such as PCR and MALDI-TOF mass spectrometry have shown great promise in identifying Mucorales, enabling much earlier and faster diagnoses [6,28]. However, these tools are still not widely available.

The treatment of ROCM remains difficult, and the prognosis depends on how quickly treatment is implemented. Treatment involves a multidisciplinary approach, combining control of the underlying pathology — particularly the management of uncontrolled diabetes — followed by surgical debridement of infected tissues and appropriate antifungal treatment [6]. Amphotericin B is the drug of choice [1,4,10,15,23,28].

## Conclusion

This case demonstrates the consequences of a delayed diagnosis and treatment of mucormycosis. Despite its rarity, this infection should be suspected in any case of facial cellulitis, especially in patients with poorly controlled diabetes, to ensure prompt and thorough clinical and laboratory management.

## Patient’s consent

Consent from the patient was obtained for the publication of this article.

## Funding

This study received no funding.

## Authors’ contributions

Imane ZOUAOUI: Study design, literature review, data collection, analysis, and interpretation, manuscript writing

Yassine BAY BAY: Conducted part of the study (performed laboratory tests, collected data)

Houda DOSS-BENNANI: Conducted part of the study (collected data, provided clinical follow-up for the patient, monitored treatment) Sarra AOUFI: Approval of the final version

## Conflicts of interest

The authors declare no conflicts of interest.
